# Supplementary material for: Reporting adverse events related to medical devices: A single center experience from a tertiary academic hospital
Source: PLoS One. 2019 Oct 24;14(10):e0224233. doi: 10.1371/journal.pone.0224233 (PMC6812847; doi:10.1371/journal.pone.0224233)
Supplement: S1 Form — (PDF) [file pone.0224233.s001.pdf]

Introduction and Consent to Participate

Intensive Care Units (ICU's) are multidisciplinary complex work environment. Efficient work flow in such areas depends on all team members' knowledge and skills, including their successful integration of new technologies in managing the critically ill patients. This technology could be either a new model of a previous medical device (like new ventilator, a new vital signs monitors, infusion and injection pumps...), or a totally new device that the ICU Staff never used before.

This survey aims to assess the level of awareness of the ICU Staff about their technical role when using new medical devices in the ICU, what factors affect their satisfaction with such new technologies, and their knowledge about the role of the technical staff (Biomedical Engineers and Technicians) in maintaining the safety of the medical devices operation.

You are invited to voluntary participate in this survey and quality improvement project, if you meet all of the following Inclusion Criteria:
- Healthcare provider (nurse, physician, respiratory therapist ...)
- Working in KSUMC for 6 months or more
- Work mainly in patients' care in acute care areas (ICUs, Emergency Department, OR)

Your participation is voluntary, and you can choose to be totally anonymous, and as recognition for your valuable time,

Thank you in advance for your valuable input, and if you have any question or comments, you can contact the Primary Investigator at dr.fahad.alsohime@gmail.com

With our Best Regards,
Dr Fahad Al-Sohime
Dr Hani Temsah
Critical Care - KSUMC

1. Consent to participate:

- ◯ I am a KSUMC Healthcare Provider (Nurse, Physician, RT...) working mainly in Acute Care area (ICU, ER, OR) AND Agree to participate
- ◯ I do NOT accept to participate

The Data Resulting From this survey WILL BE CONSIDERED Anonymous

2. Discipline/Area

3. What is your role in the ICU:

4. What is your gender?

- Female
- Male

5. For how long you have been working in the ICU?

- 1-2 years
- 3-5 years
- 6-10 years
- More than 10 years

Please answer the following from your actual practice:

6. Are you aware of the PPM (Periodic Preventative Maintenance) for medical devices in your ICU?

- ◯ Yes
- ◯ No

7. Who performs PPM in your ICU?

- ☐ Nurses
- ☐ Physicians
- ☐ Biomedical Engineering
- ☐ Vendors (Company)
- ☐ Other (please specify)

* 8. **On a Scale from 1 to 5, can you determine your level of comfort in dealing with the following devices in the ICU?**
(1: not at all comfortable to 5: completely comfortable)

	1	2	3	4	5
Infusion Pumps	◯	◯	◯	◯	◯
Mechanical Ventilator	◯	◯	◯	◯	◯
Non Invasive BP monitoring	◯	◯	◯	◯	◯
pulse oxymeter	◯	◯	◯	◯	◯
ECG	◯	◯	◯	◯	◯
Defebrillator	◯	◯	◯	◯	◯

9. **Regarding the Following Medical Devices, Can you determine:**

	Did you receive formal training to this device	Is there a super user for this device	Is the user manual reachable	How do you learn more about the device
Infusion Pump "Volumat MC Agilia"				
Mechanical Ventilator " Servo i"				
Cardiac Monitor "Carescape"				
ECG "Mac 5500"				
Defebrillator "Heart Stream XL"				

10. **In the past year, Have you ever faced a Technical problem with any of the previously mentioned machines such as User Error, device malfunction or sudden shut down of the machine**

- Yes
- No

* 11. **When there is an alarm from one of the medical devices, how do you trouble shoot these issues?**

Arrange your approach steps according to the priority from 1 to 6.

- I Respond to it appropriately
- Contact the super user or the senior
- Call the Biomed technician
- Call the company
- I turn it off
- Other

12. **In your center .The following procedure regarding medical devices, are performed by whom?**

	Nurse	Head Nurse	Super user	Physician	Biomed Technician	The manufacture
Equipment assembling						
Trouble shooting						
Oerder Supply						
Maintenance & device calibration						
Sterilization						
Disposals						

* 13. **How do you get update regarding the potential adverse events of different medical devices ?**

- [ ] Manufacture,
- [ ] Ministry of health,
- [ ] Public advertisement,
- [ ] SFDA,
- [ ] Medical Literature
- [ ] Colleagues
- [ ] Other (please specify)

14. **If an adverse event related to a medical devices, Are you aware about any official national reporting system regarding these events?**

- ( ) Yes
- ( ) No

Troubleshooting / reporting

15. **If Yes, What is the organization that you will report to? (Choose all that apply)**

- [ ] Ministry of health
- [ ] Saudi Council for Health Specialities
- [ ] Saudi Food and drug organization
- [ ] The council of cooperative health insurance
- [ ] CBAHI
- [ ] Other (please specify)

16. **How do you report these Adverse events? (You can choose more than one answer)**

- [ ] Website
- [ ] Email
- [ ] Phone
- [ ] Fax
- [ ] Other (please specify)

17. **Have you ever reported an adverse event to this system?**

- ○ Yes
- ○ No

Copy of page: **The Data Resulting From this survey**

18. **If yes, did you receive feedback regarding your report?**

- ◯ Yes
- ◯ No

19. **How much were you satisfied with feedback you received? Why?**

Not satisfied	Minimal satisfaction	Mildly satisfied	Satisfied	Extremely satisfied
☆	☆	☆	☆	☆

Reason for your answer above:
