# Supplementary material for: Reporting adverse events related to medical devices: A single center experience from a tertiary academic hospital
Source: PLoS One. 2019 Oct 24;14(10):e0224233. doi: 10.1371/journal.pone.0224233 (PMC6812847; doi:10.1371/journal.pone.0224233)
Supplement: Attachment — Submitted filename: Reviewer reply 2 Version 1 21Sep2019.docx [file pone.0224233.s004.docx]

Dear Dr. Kamolz

We would like to thank you and the reviewers for the comments and suggestions pertaining to the manuscript #PONE-D-19-19700: “Reporting adverse events related to medical devices: A single center experience from a tertiary academic hospital” in PLOS ONE

We have now addressed all points, as detailed below.

**Reviewer #1: Thank you for the revision.**

**Major comments**

1- I am not familiar with the type of statistics used in the manuscript. The fixed effects of the types of ICUs may need to be controlled or specified by the ICUs, because the types of medical devices most frequently used and the levels of training might vary accordingly.

Thanks dear reviewer for the above comment. Regarding the first part, we suggest to refer to the below studies which can explain the type of the statics that we applied for our study.

For the second part in term of fixed effects of the types of the ICUs, we conducted a descriptive, qualitative and quantitative analysis for universal parameters and concepts applicable for the different types of the ICUs. Investigated in this study.

Aziz, N., Zain, Z., Mafuzi, R. M. Z. R., Mustapa, A. M., Najib, N. H. M., & Lah, N. F. N. (2016). *Relative importance index (RII) in ranking of procrastination factors among university students.*

Holt, G. D. (2014). *Asking questions, analysing answers: relative importance revisited. Construction Innovation, 14(1), 2–16.*

2- Discussion: there are several recommendations. However, they are not all based on the findings. For example, “The reporting system of adverse events to the SFDA should be anonymous, confidential, and non-punitive” was unrelated to the findings and inferred based on the comparisons with other jurisdictions. The non-punitive principle was mentioned in the first and second points. The term, “activated”, was not clear.

Thank you for your observation, adjustments carried out as it appears in the below text and these changes were done in the manuscript.

Many thanks

**Conclusion and Recommendations**

This study has raised the need to develop a framework for the safe operation of medical devices, based on international standards, that has the following characteristics:

- The Health care workers need to be familiar with the existence of a national reporting system of adverse events to the SFDA. Legislation that does not penalize the reporter should be created.
- The role of the Medical Devices SFDA-Officer for healthcare providers should be activated (Saudi Food & Drug Authority 2018).
- The internal E-OVR in the hospitals should be directed to the SFDA.
- Recalls for medical devices should be directed from the SFDA through the Saudi Center for Patient Safety and disseminated to hospitals.
- A database should be created where risk information is categorized and made accessible with multiple interfaces for:
  - Medical device users (Voluntary) – the aim of this database is to facilitate networking and sharing experiences among users
  - Medical device manufacturers or designers (mandatory)
  - Healthcare organizations (such as for risk management departments) (mandatory).

**Minor comments**
1- Results: please use the tense consistently. Both present and past tenses were used.

Thank you for your remark, changes done accordingly in the text.

2- Abstract: “international standard”? Do you mean the Danish system?

Mainly we refer to the standards implemented in the western countries such as the United States Food and Drug Administration (USFDA) or the Danish Health Care System approach.^16-17^

3- Table 1 and 2: please add total.

Indeed, we added the total

4- Fig 1 & 2: the units? Abbreviations?

Thank you for your kind observation. Changes carried out.

**Figure 1:** Nurses reported destinations of reporting equipment related adverse event alerts/incidence.

**Figure 2:** Nurses Sources of update Information on equipment adverse occurrence alerts.

**Reviewer #2:**

thank you for your revision. In my opinion, there is still some space for improvement. I would like to ask you to consider the following:
1.) While I can imagine, that the development of the MDNR system remains in its infancy because it has just been amended, it still remains elusive to me, if you mean that. If so, please indicate (see previous comment 1.)

Thank you for your notion. Exactly, as the national law of “ Medical Devices Interim Regulation” was Issued by the SFDA Board of Directors on 27 December 2008, and amended on 27 December 2017 (change in the text carried out)

And this was elaborated from Ref 4 too that states in their abstract: “However, the progress of medical device registry systems and post-market medical device surveillance systems remains in its infancy in Saudi Arabia and within the region. In 2007, a royal decree assigned the responsibility for regulating medical devices to the Saudi Food and Drug Authority (SFDA). Soon afterwards, the SFDA established the Medical Devices National Registry (MDNR) to house medical device information relating to manufacturers, agents, suppliers and end-users.

2.) Even if you want to elaborate more on the adverse events in the discussion, the logical flow is compromised if you switch the orders. If you want to discuss the personal experience before the approach, please switch the order in the methods section then. (see previous comment 3)

  Thank you for this remark. We totally agree with you and we switched the order in the method section.

3.)In table 2, the third question still makes no sense to me. How can the frequency of no be 253, if the whole n is just 102? Again a typo? Should it be 58? (See previous comment 7).

Our sincere apology, that was a typing error.

The question “**Have you ever reported an adverse occurrence failure of any equipment?”** Was directed to all the nurses, 44 of them indicated ‘yes’, and 253 answered ‘No’

4.) Even if you explained the 2% (6 out of 279) now, you should really introduce that number in the results already. I do not want to think about the numbers again, when I am in discussion already. Every "new" result just leads to confusion. (see previous comment 12).

Indeed, This was moved to the Results section, thank you for the advice.

5.) You should really introduce a clearly delineated limitations section. (see previous comment 11)

This is an excellent advice. It was added to the text

**Study limitations:**

We considered the usage error as one of the possible technical problem that might be faced by the healthcare worker as this might result from poor usability research and design by the manufacturer. The usability and human factor issues are considered sources of hazards that need to be eliminated or controlled based on the International Electrotechnical Commission (IEC) requirements. ^1^

While this study highlights significant medical device reporting system from one hospital, this needs further exploration in the other healthcare facilities. The self-reporting nature of this survey may be subjective to recall bias that needs further direct observations in prospective, ICU-based clinical trials.
