# Supplementary material for: Reporting adverse events related to medical devices: A single center experience from a tertiary academic hospital
Source: PLoS One. 2019 Oct 24;14(10):e0224233. doi: 10.1371/journal.pone.0224233 (PMC6812847; doi:10.1371/journal.pone.0224233)
Supplement: Attachment — Submitted filename: Reviewer comments 24-8-2019.docx [file pone.0224233.s003.docx]

Dear Dr. Kamolz

We would like to thank you and the reviewers for the comments and suggestions pertaining to the manuscript #PONE-D-19-19700: “Reporting adverse events related to medical devices: A single center experience from a tertiary academic hospital” in PLOS ONE

We have now addressed all points, as detailed below.

Reviewer #1: Reviewer’s comments

A reporting system was described in this manuscript. However, there are some issues that need to be addressed.

Major comments

1. There is little description about the national reporting system, the name of the law, the time of legislation, the details in the required reporting, etc.

Thank you for your comment. The name of the national law is the “Medical Devices Interim Regulation“. This law was issued by the Saudi Food and Drug Authority (SFDA) Board of Directors decree number (1-8-1429) and dated 27 December 2008, and amended by SFDA Board of Directors decree number (4-16-1439) dated 27 December 2017

(<https://www.sfda.gov.sa/ar/medicaldevices/regulations/Documents/MD-InterimRegulation-en.pdf>)

The details in the required reporting can be found in the folowing link

(<https://ncmdr.sfda.gov.sa/Default.aspx>)

Changes done in the text.

2. Demographic characteristics: the nationality/citizenship was not reported. Howe many of them were native? Do they have difficulties in understanding the regulations?

Thank you for raising this point. We did not enquire about the nationality to avoid creating bias among the healthcare workers. All of them speak English fluently as first or second language. The questionnaire was constructed in English knowing that the official language of the healthcare system in Saudi Arabia is the English language and all the legislative regulations related to the healthcare are issued in both English and Arabic.

3. Can the authors be more specific about the “devices”? What are the devices that are more frequently used in the settings?

Thank you for this comment. The medical devices indicated in the questionnaire were the devices related to the critical care settings; such as mechanical ventilators, defibrillator, cardiopulmonary monitors, infusion pumps,.. etc.

Changes done in the text.

4. How was “Respond to it appropriately by examination” in the questionnaire defined?

Thank you for your comment. Appropriate response was defined as “examining the device as per the manufacturer‘s guidelines (User Manual) as received during the user training on a particular device“.

Changes done in the text.

5. The reporting interface (?) or methods were not described. In the Results, it seemed complicated. Was the reporting part of nursing education?

Thank you for your point. Indeed, the reporting interface is an electronic website (<https://ncmdr.sfda.gov.sa/Default.aspx>) designed for healthcare workers to report the adverse events. Unfortunately, it is not included in the nursing education and this study aimed to support the need to integrate this reporting system the healthcare education.

6. In the Discussion, the explanations about the key terms should be moved to the Introduction or the Methods.

Thank for your comment. In the beginning of the discussion, we wanted to elaborate more on the adverse events related to medical devices as this would help the reader to better understand the evolution of this system over the last several years.

Minor comments

1. In the first sentence, Line 67, what does “(a 95% confidence interval and 5% marginal error)” mean?

Sorry for the typo, it was corrected to:

“95 % confidence and a margin of error equal to 5%”

The 95% level is the most commonly used. If the level of confidence is 95%, the "true" percentage for the entire population would be within the margin of error around a poll's reported percentage 95% of the time.

<http://theknowledge.site/wp/m/Margin_of_error.htm>

2. In the Discussion, was there any data about the reporting rates in previous studies?

Unfortunately, studies in this field are scarce and extremely limited. The existing source of the reporting rate of adverse events related to medical device over the last several years is the annual statistical report released by the Saudi Food and Drug Authority SFDA

Reviewer #2: Dear authors,

I got to review your manuscript "Reporting adverse events related to medical devices: A single center experience from a tertiary academic hospital". In my opinion, your manuscript is already in a quite good state dealing with a topic highly worthwhile to further assess. However, there are some aspects, that should be covered/questions that were raised during the reading:

1.) In your introduction, line 53, you state that the development of the MDNR system remains in its infancy.

However, you do not further elaborate. Please write one or two sentences as to why this system is not that well developed yet.

Thank you for your comment. This was elaborated now in the text, mentionioning that the national law “ Medical Devices Interim Regulation“ was Issued by the Saudi Food and Drug Authority Board of Directors decree number (1-8-1429) and dated 27 December 2008, and amended by Saudi Food and Drug Authority Board of Directors decree number (4-16-1439) dated 27 December 2017

(<https://www.sfda.gov.sa/ar/medicaldevices/regulations/Documents/MD-InterimRegulation-en.pdf>)

2.) In line 71 you state, that the questionnaire was drafted by the authors based on a review of the literature - can you further elaborate as to how this review was assessed? Do you refer to specific papers?

A combination of evidence appraisal together with expert opinion were used in order to draft a suitable questionnaire for this study.

MEDLINE® and PubMed searches were performed using the search terms “medical”, “device”, “reporting”, “safety” “ Usability” and “‘adverse events OR critical care’”. The reference lists of identified papers were screened to identify other relevant papers.

*Teow N and Siegel SJ. FDA Regulation of Medical Devices and Medical Device Reporting. Pharmaceut Reg Affairs. 2013;2:110.

* Geissler N, Byrnes T, Lauer W, Radermacher K, Kotzsch S, Korb W, Hölscher UM. Patient safety related to the use of medical devices: a review and investigation of the current status in the medical device industry. Biomed Tech (Berl). 2013;58(1):67-78.

* Mattox E. Medical devices and patient safety. Crit Care Nurse. 2012;32(4):60-8.

* Rich S. How human factors lead to medical device adverse events. Nursing. 2008 Jun;38(6):62-3.

3.) Please keep the same order in Methods (1, 2, 3) and in results/discussion (1, 3, 2). I recommend to first present the results of the step sequence and THEN the personal experience (as it is in methods section).

Thank for your comment. In the beginning of the discussion, we wanted to elaborate more on the adverse events related to medical devices as this would help the reader to better understand the evolution of this system over the last several years.

4.) Have there been any nurse pupils, assistance care givers been questioned, as well?

No, in our study we included only the practicing nurses.

5.) Please delete "approximately" in line 118, as it makes no sense.

Thanks for highlighting this, it was deleted in the revised manuscript.

6.) I recommend to give the percentage of those who had reported an AE compared to those who were aware of the system, as you state in the sentence (line 121f.). Then you can also lose the next sentence.

The aim of the study is to explore the awareness of the correct national reporting system, that’s why the question about if they ever reported an adverse event or not was limited only to the 102 nurses who reported that they were aware of the national reporting system as we assumed that those nurses who were not aware about the reporting system were never exposed to an event that lead to report this event. So only 44 nurses (14.8%) among the 102 nurses who were aware of the reporting system had reported at least one equipment-related adverse event over the last year.

7.) In table 2, why does it say n=103 at the third question? Does that not have to be 102? And further, if n=102, how come the frequency is higher? I would only indicate the nurses, that have ticked yes in the second question (therefore n=102; yes: 44, No: 58).

Thank you. Indeed, this is a typing error. Correction carried out

8.) In your sequence of steps, I do miss the relevance of the patient - how comes, that there is no step "securing the patient's survival" or similar?

Thank you for this important point. The patient safety is always included in the Appropriate response to a device malfunction as per the manufacturer guidelines (User Manual). Changes done in the text.

9.) In table 3 the n at "call the biomed technician" is missing.

The typo was corrected.

10.) On page 12 in your discussion, you state, that the nurses had also reported usage errors - however, prior to that there is no mentioning of usage errors, just technical errors. So I recommend to delete it here AND
11.) Introduce a limitations section, in which you could also discuss, that some of these technical errors could be due to wrong usage and so on. In general, a limitations section would be helpful.

Thank you. We considered the usage error as one of the possible technical error that might be faced by the healthcare worker and this was indicated in the questionnaire. Correction carried out in the text.

We considered the usage error among the technical problems as poor usability research and design by the manufacturer, which could result in recall of the product because usability and human factor issues are considered sources of hazards that need to be eliminated or controlled based on the International Electrotechnical Commission (IEC) requirements

12.) In the middle of page 14, where do the 2% come from? I thought it would have been n=102, therefore around 34%? If the rest could not name the right system, then please introduce these 2% and this fact in results section already.

6 respondents out of 279 respondents considered the Saudi FDA the source of risk information such as recalls.

13.) Have you found any correlation between length of experience/age with the knowledge of the existence of that database? Please add this to your manuscript.

No correlation has been found. This information has been added to the text

14.) Have you found any literature concerning the same topic with other medical professions? E.g. doctors - I would be interested in how many of those know about the existence of said systems. Please discuss.

Unfortunately, studies in this field are scarce and extremely limited.

We included the nurses in the critical areas as they are the frontliners who are dealing first with these technical issues
